# Mesenchymal Stromal Cell Therapy in the Management of Perianal Fistulas in Crohn’s Disease: An Up-To-Date Review

**DOI:** 10.3390/medicina56110563

**Published:** 2020-10-27

**Authors:** Gaetano Gallo, Vincenzo Tiesi, Serena Fulginiti, Gilda De Paola, Giuseppina Vescio, Giuseppe Sammarco

**Affiliations:** 1 Department of Medical and Surgical Sciences, University of Catanzaro, Viale Europa, 88100 Catanzaro, Italy; vincenzotiesi@libero.it (V.T.); serena.fulginiti@hotmail.it (S.F.); gilda_depaola@libero.it (G.D.P.); vescio@unicz.it (G.V.); 2 Department of Health Sciences, University of Catanzaro, Viale Europa, 88100 Catanzaro, Italy; sammarco@unicz.it

**Keywords:** mesenchymal stromal cells, stem cells, Crohn disease, perianal fistula, fistulizing CD, surgical treatment

## Abstract

Crohn’s Disease (CD) is a chronic inflammatory disorder that potentially involves the entire gastrointestinal tract. Perianal fistulizing CD (pCD) is a serious and frequent complication associated with significant morbidities and a heavy negative impact on quality of life. The aim of CD treatment is to induce and maintain disease remission and to promote mucosal repair. Unfortunately, even the best therapeutic regimens in pCD do not have long-term efficacy and cause a significant number of side effects. Therefore, it is mandatory to study new therapeutical options such as the use of mesenchymal stromal cells (MSCs). These cells promote tissue repair via the induction of immunomodulation. The present review aims to analyze the existing updated scientific literature on MSCs adoption in the treatment of pCD to evaluate its efficacy and safety and to compare the use of bone marrow and adipose tissue derived MSCs, type of administration, and dose required for recovery.

## 1. Introduction

Crohn’s disease (CD) is a chronic inflammatory disorder that may potentially involve any portion of the gastrointestinal tract. Most patients initially complain of significant mucosal inflammation. Unfortunately, over time, disease behavior can change, and many patients progress to penetrating complications, with a full thickness involvement of the gut wall, including sinus, abscess and fistulas [1,2,3,4]. The transmural inflammation predisposes CD patients to fistula formation, but the first step in this process is generally assumed to be tissue destruction. In this context, several studies suggested the role of epithelial-to-mesenchymal-transition (EMT) as a driving force behind the development of fistulizing CD being TGF-β the principal inducer [5,6].

Perianal Fistula in CD (pCD) were first described in 1938 as a typical complication of terminal ileitis and, as demonstrated by several population-based studies, at least one third of CD patients will develop fistulas over the disease’s course [5,7]. Perianal fistula development depends on the localization of CD. Frequently, it is associated with colonic and rectal involvement (92% of patients), while it is rare in patients with isolated ileitis (12% of patients) [8,9].

Perineal fistulizing CD develops in the 20% of patients and typically shows a relapsing and remitting course [5]. It is possible to identify high levels of tumor necrosis factor (TNF), IL-13 and TGF-β all around the fistula. The presence of these factors supports the hypothesis of intestinal microbiota and EMT active role in the pathogenesis of the disease. Fistulization is a negative predictor of long-term disease and its treatment requires a multidisciplinary approach. In this context, the use of mesenchymal stromal cells (MSCs) is attracting the interest of scientific community [10].

## 2. Mesenchymal Stromal Cells

MSCs are multipotent cells capable of self-renewal and differentiation. In particular, the Mesenchymal and Tissue Stem Cell Committee of the International Society for Cellular Therapy (ISCT) identified the three minimal criteria to define MSCs: (1) adherence to plastic; (2) expression of specific surface antigens such as CD105, CD73 and CD90; (3) and the ability to differentiate in standard vitro tissue-culture condition into the main trilineages of mesenchymal derivation or rather osteoblast, adipocytes and chondroblasts [11]. MSCs have been isolated in various tissues such as circulating blood, umbilical cord or placenta, but the main donors are adipose tissue and bone marrow [12,13,14,15]. However, within adipose tissue, it is possible to extract a greater number of MSCs, or adipose-derived stem cells (ASCs), than those isolated in the bone marrow [16].

The methods of extraction of ASCs keep changing over time. In this regard, Cytori Therapeutics Inc. (San Diego, CA, USA) created the Celution^TM^ system, or rather a closed system capable of extracting ASCs from subcutaneous tissue. The extraction involves several steps including the use of collagenases at a concentration of 0.075% and short centrifugation cycles until a minimum volume of ready-to-use MSCs is extracted, normally within 1 h [17].

Lipogems^®^ technology was first described in 2010, given the need to have a minimally manipulated product with a shorter production time.

In fact, a small amount of fat tissue is harvested from the donor site and it is subsequently processed using the Lipogems^®^ device, being ready for clinical use after less than 20 min. The latter represents a great advantage especially if compared with the time required for enzymatic digestion.

Lastly, it preserves an intact stromal vascular niche due to the use of slight mechanical forces [18]. However, despite the many advantages, in patients with a low BMI, such as those with CD, the lower amount of stem cells extracted with this method could be a problem in term of efficacy.

**Figure 1:**
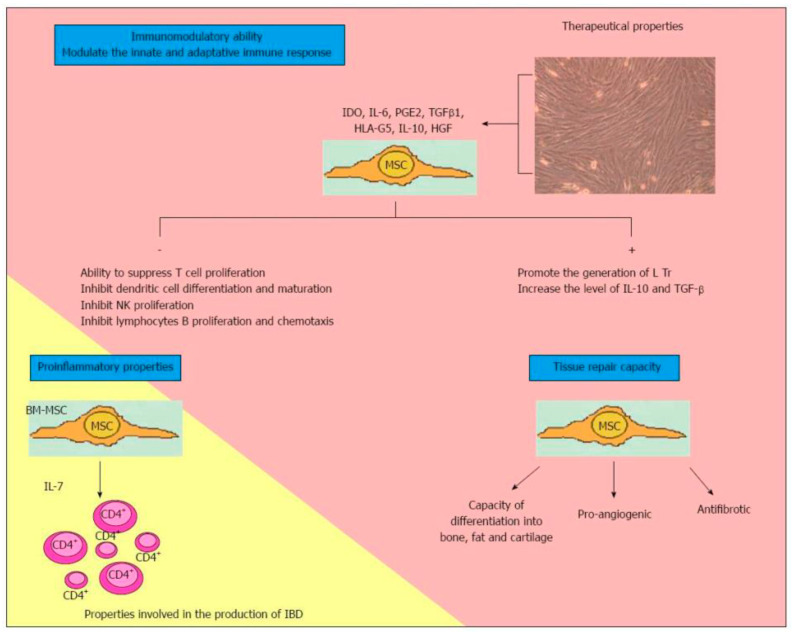
Characteristics of multipotent mesenchymal stromal cells. MSCs: Multipotent mesenchymal cells; IDO: Indoleamine 2,3 dioxygenase; IL: Interleukin; PGE2: Prostaglandin E2; TGFβ1: Transforming growth factor beta-1; HLA-G5: Human Leucocyte Antigen G5; HGF: Human growth factor; NK: Natural killer; Tregs: Regulatory T cells; BM-MSC: Bone-marrow-mesenchymal stem cell; CD4+: Activated lymphocytes CD4; IBD: Inflammatory bowel disease. Reproduced with permission from Martínez-Montiel MDP et al. [19].

The use of MSCs for the treatment of pCD seems to be justified by their immunomodulatory effect (Figure 1) [19]. This latter lies in the ability of MSCs to upregulate a subset of TCD4 + cells of regulatory T cells (Treg) [20,21]. In fact, a decrease in Treg cells and the coexistence of an altered ratio between Treg cells and T effector cells represent the basis of CD pathogenesis [22,23]. MSCs, therefore, can reduce the immune response both through the upregulation of the Treg, migrating in the inflammation sites and also determining regeneration, and the healing of damaged tissues [24,25]. Nevertheless, their immunomodulatory function can be achieved through the involvement of several other molecules and patterns, such as B-cell proliferation or production of CXCL9, CXCL10 and CCL-2. In fact, they assume a proinflammatory or immunosuppressive phenotype based on the TRL signals received [21].

The present study aims to report the current evidence on the efficacy and safety of MSCs in the treatment of pCD.

## 3. Surgical Management

The treatment of pCD still represents a clinical challenge; in this context, surgical treatment represents an essential step for both definitive healing and infection control. The choice of the most appropriate surgical technique depends on different factors such as the anatomy of the fistula, the presence of local inflammation and the level of experience of the surgeon [26,27,28,29,30].

Among the most commonly adopted surgical techniques, the use of a draining seton allows a bridge to surgery, whereas the use of a cutting seton on one side may permit fistula healing, but on the other one may cause faecal incontinencein in 5–10% of patients [30,31].

The most used sphincter-saving procedure is represented by the endorectal advancement flaps, but when used in fistulas associated to CD, it shows a lower rate of success in comparison with fistulas of cryptoglandular etiology [32,33,34,35]. Other sphincter-saving approaches such as the use of fibrin glue [36,37], Fistula-Tract Laser Closure (FiLaC) [30,38], Ligation of Intersphincteric Fistula Tract (LIFT) [39] or Video-Assisted Anal Fistula Treatment (VAAFT) [38,40] can be considered, but their success rate remains low, especially in patients with uncontrolled pCD. As far as refractory pCD is concerned, a temporary or definitive faecal diversion can be considered and discussed with the patients [39,41].

## 4. State of the Art

### 4.1. Mesenchymal Stromal Cells in Perianal Fistulizing Crohn Disease

The progenitor cells were identified in the bone marrow about a century ago, and the observations of Friedenstein and colleagues, obtained through the isolation of cells adhering to plastic or colony-forming unit fibroblasts, allowed Owen first and Caplan later to define these colonies as “mesenchymal stem cells” in 1980 [42,43,44,45,46]. They consist of only 0.001% to 0.01% of the total nuclear cells, representing a lower percentage if compared with the hemopoietic stem cells [47,48].

Although most studies about MSCs have been carried out on cells derived from bone marrow, cells deriving from muscular tissue, adipose tissue, blood and umbilical cord are also under examination [49].

In addition to the ability to support hemopoiesis in the bone marrow [50], MSCs are able to promote tissue repair and inflammation control in situ via some cellular signals which are still not fully understood—in particular, both the insulin-like growth factor and the angiopoietin-1 recruit macrophages and fibroblasts, which are essential in the production of collagen.

Among the immunomodulatory characteristics of MSCs, three steps were identified:

1. Their migration into the inflammation site or tissue injury [51];
2. The secretion of anti-inflammatory molecular factors such as Interleukin-10 (IL-10), HGF, TGFβ1 and Indoleamine 2,3-dioxygenase (IDO) [52,53];
3. The maintenance of the local anti-inflammatory effect by sending paracrine signals to neighboring cells [54,55].

These immunomodulatory properties, influencing the profiles mentioned and modulating immune cells such as lymphocytes, dendritic cells and macrophages, allowed their therapeutic use in serious diseases such as Graft versus Host Disease (GvHD) [56,57], systemic lupus erythematosus (SLE) [58], myocardial infarction [59], multiple sclerosis [60], COronaVIrus Disease (COVID-19) [61,62], enterocutaneous fistulas [63] and CD [64], even if the exact therapeutic mechanism is not yet known.

### 4.2. Literature Review

The first Phase I study carried out to demonstrate the efficacy and safety of autologous stem cell treatment for pCD was conducted by Garcia-Olmo et al., 2005. This prospective study involved five patients (three males and two females) but due to gram-positive bacterial contamination of cultured cells, one patient was excluded. A total of nine fistulas (one suprasphincteric, three rectovaginal, five enterocutaneous) were locally inoculated with 3 × 10^6^ ASCs from lipoaspirate, and eight fistulas were able to continue the follow-up at 8 weeks, as the patient with enterocutaneous fistula was excluded due to emergency abdominal surgery. Of the remaining eight fistulas, six achieved complete healing (75%); the other two fistulas achieved partial healing with reduced external drainage. No adverse effects were detected and, to confirm the safety and feasibility of treatment with ASCs, histopathological analisis were carried out and showed that there was no cytological transformation (Table 1) [64].

However, even if no cases of malignant transformation after perianal treatment with MSCs were registered, these cells could have protumorigenic abilities on tumors by promoting the proliferation of neoplastic cells and neoangiogenesis. Therefore, further studies with long-term follow-up are necessary [65,66,67].

Local injection demonstrated a superior therapeutic efficacy respect to systemic administration. In fact, the systemic use does not allow the migration of a sufficient number of MSCs towards the inflammation site [68,69,70,71,72,73,74]. Moreover, local treatment of MSCs combined with fibrin glue resulted in the healing of 71% of pCD cases, compared to the injection of fibrin glue alone, which was efficacious in 16% of pCD cases [75].

Garcia-Olmo [75,76] first, and Molendijk [77,78] later, proposed a standardized method for the local injection of MSCs in pCD. According to the latter, the use of MRI and examination under anesthesia to locate the fistula is mandatory. Following the drainage of the abscess, a loose seton should be placed and patients with proctitis should also start medical therapy. Before local injection, and after the removal of the seton, the fistulous tract is curetted, and the internal orifice closed. MSCs should be injected around the internal orifice, and also through the external one, avoiding direct injection into the lumen in order to not waste therapeutic agents.

Those results were confirmed by an observational study published by Garcia-Olmo and colleagues concerning the treatment of enterocutaneaous fistulas in patients with CD [79]. In particular, a complete re-epithelialization was observed in 75% (3 out of 4) of fistulas treated with expanded ASCs compared to 25% (1 out of 4) of those treated with the not-expanded stromal vascular fraction (SVF) directly from the lipoaspirate sample.

After the extraction and expansion, ASCs are more genetically and morphologically stable and show greater proliferative capacity and angiogenetic properties [80,81,82]. Furthermore, the greatest strength is represented by the superior expression of tissue growth factor, reducing hemocompatibility [83].

There are no trials comparing autologous and allogeneic MSCs. It is evident that the expansion of autologous MSCs in vitro takes several weeks and, before administration, there are several preparation phases with a greater risk of loss of stem cells. Nevertheless, cell quality is also affected by patients’ disease status [84,85,86].

To date, the European Medicines Agency (EMA) has approved Cx601-Darvadstrocel (Alofisel^®^) for the treatment of refractory pCD in adult patients with inactive/mildly active luminal CD who have shown no response to the first-line approach with conventional or biological therapy [87,88,89]. Darvadstrocel is a preparation of human allogeneic expanded adipose-derived stem cells (eASCs) [90,91].

Darvadstrocel is administered in single-dose by intralesional injection of 4 vials of 120 million in 24 mL after curettage of the fistulous tract and closure of the internal orifice [90]. Up to three fistulous tracts can be treated, but its use is only approved for clinical trials and compassionate use programs regulated by the European Medicines Agency [90,92].

Herreros et al. in 2019 evaluated the efficacy of compassionate use of ASCs (autologous eASCs, allogenic eASCs and SVF) in the treatment of 42 perianal fistulas and a total of 52 cases including 7 rectovaginal, 1 sacral, 1 urethrorectal fistula, and 1 suppurative hidradenitis. In particular, 18 perianal fistulas were related to CD. Partial improvement or complete healing were observed in 49 out of 52 cases (94.2%) in almost 6 weeks. Conversely, considering only CD-associated fistulas, 100% (18/18) experienced improvement or complete recovery in a mean time of 5.3 weeks, and healing was achieved in 10 out of 18 cases (55.5%) [93].

The most important study that has evaluated the efficacy and safety of ASCs in pCD is the Adipose-Derived Mesenchymal Stem Cells for Induction of Remission in Perianal Fistulizing Crohn’s Disease (ADMIRE-CD), a phase III, randomised, double-blind, parallel group, placebo-controlled study [68]. The primary outcome of the ADMIRE-CD study was to assess the closure of external draining openings and the absence of collections >2 cm observed on MRI after 24 weeks [68,94].

The patients selected in the study were aged >18 years, with inactive or slightly active disease, Crohn’s Disease Activity Index (CDAI) of 220 or less, with at least two and a maximum of three external openings and with up to two internal openings.

Patients were randomized (1:1 ratio) in two groups: the active group (*n* = 107) who received a single injection of Cx601, a 24 mL solution with 120 million of ASCs, two weeks after courettage of the fistula and, possibly, after the placement of a draining seton removed at the time of injection; the placebo group (*n* = 105 patients), in which the same volume of saline solution was administered. The 24 weeks follow-up was completed by 171 out of 212 patients (81%).

The primary endpoint was achieved in the Intention-to-analysis (ITT) by 53 out of 107 patients (49.5%) in the Cx601 group, and by 36 out of 105 (34.2%) in the placebo group. These results were consistent with those reported in the modified ITT (mITT) analysis, in which 53 out of 103 (51.4%) patients of the active group and 36 out of 101 (35.6%) patients of the placebo group achieved the primary outcome [68]. A total of 131 patients (61.8%) completed the 52-week follow up and the mITT analysis showed a combined remission rate of 56.3% (58/103 patients) in the Cx601 group and 38.6% (39/101 patients) for the placebo group, while a clinical remission was observed in 59.2% of patients in comparison to 41.6% of the control group [94].

The most common treatment-related adverse events were anal abscess (six in the Cx601 group and nine in the placebo group) and proctalgia (five in the Cx601 group and nine in the placebo group) [68]. After 52 weeks, there was a higher rate of adverse events in both groups (76.7% in the Cx601 group and 72.5% in the placebo group). Around 8.7% patients experienced a side effect which led to study withdrawal, while most of the patients referred mild to medium events such as nasopharyngitis, diarrhoea, pyrexia, arthralgia, abdominal pain, flare of Crohn’s disease. According to the authors, one of the most important findings was the maintenance of efficacy for up to one year [94].

Furthermore, in ADMIRE-CD, clinical recovery has been reached in 59.2% patients at 52 weeks, whereas in ACCENT II study (A Crohn’s Disease Clinical Trial Evaluating Infiximab in a New Long-term Treatment Regimen in Patients With Fistulizing Crohn’s Disease), recovery rate was 36% at 54 weeks [95]. In fact, the ACCENT II study is a randomized, double-blind, placebo-controlled study that evaluated the efficacy and safety of infliximab treatment for the maintenance of rectovaginal fistula’s closure in patients with fistulizing CD through intravenous infusions of 5 mg/kg of infliximab, the success rate, and thus the fistula’s closure.

These data highlighted the need for new trials on infliximab treatment in combination with eASC to evaluate the possible superiority of their combination [94] and were consistent with those described by other phase I-II trials [75,96].

In the United States, a similar study, the so called ADMIRE-CD-II (https://clinicaltrials.gov/ct2/show/NCT03279081), is underway, with the aim of demonstrating the efficacy of darvadstrocel in the treatment of complex PF in patients with CD and 600 patients have been enrolled to date [91].

In Europe, there is a postmarketing registry called INSPIRE, an observatIoNal poSt-marketing registry on the effectiveness and safety of darvadstrocel in PatIents with CRohn’s disease and complex pErianal fistulas. This registry aims to establish a framework for acquiring real-world data about the efficacy and safety of the commercially available local MSC therapy. Collected data will soon be available [97].

Recently, Dietz at al. described the effect of autologous ASCs after extraction, cryopreservation and thawing at the time of use. After 6 months, 10 out of 12 (83%) patients showed complete healing and only one patient had abscess formation [98]. Dige et al. [99], however, in a study with 21 patients directly injected the adipose tissue containing the autologous ASCs and demonstrated their effectiveness.

A further study was conducted by Zhou et al. in 2020. Twenty-two patients were enrolled in an open-label, randomized, controlled clinical trial and the safety and efficacy of treatment with ASCs was compared with the incision-thread-drawing procedure. Follow-up was performed at 3 months, 6 months, and 12 months, although the latter was completed by 17 out of 22 patients. The primary endpoint was fistula’s closure and was achieved in both groups with no substantial differences: 10/11 (90.9%) vs. 5/11 (45.5%) at 3 months; 8/11 (72.7%) vs. 6/11 (54.5%) at 6 months; and 7/11 (63.6%) vs. 6/11 (54.5%) at 12 months. Nevertheless, there was a statistically significant superiority of the ASCs group in terms of improvement of the secondary endpoints (simplified Crohn’s Disease Activity Index (CDAI), Perianal Disease Activity Index (PDAI), pain scores with Visual Analog Score (VAS), Inflammatory Bowel Disease Questionnaire (IBDQ), and Wexner score). Neither adverse events nor incontinence problems occurred in both groups, even if sphincter function has been shown to be better in the ASCs group [100].

A recently published meta-analysis reported that the percentage of complete healing of perianal fistulas in CD using MSCs is 64.1% (95% CI 52.3–74.5), which is a higher percentage than the one observed in cryptoglandular fistulas (61.5%; 95% CI 36.8–81.4). There are elements of heterogeneity that limit the execution of a noteworthy meta-analysis, since the numerous studies conducted to date have used different type of MSCs, different origins, different amounts and different injection methods. Despite these limitations, it seems that MSCs can be considered a valid alternative for the treatment of both CD and non-CD fistulas and autologous MSCs are able to achieve higher healing rates than allogenic MSCs. The outcomes were favorable when the amount of MSCs injected is proportionate to the size of the fistula [101].

An interesting application of ASCs concerns the treatment of pCD in pregnant women. In a recent retrospective study, the possible influence of intralesional therapy with ASCs (autologous and/or allogenic) on fertility and fetal development was investigated. Six women, with a mean age of 34.4 years during therapy with ASCs (from a minimum of 17 months to a maximum of 6 years), decided to become pregnant and were included in the study. One patient was lost at follow-up and was excluded from the analysis. One patient had both rectovaginal and perianal fistulas, two patients had only rectovaginal fistulas and two patients had only perianal fistulas. At the end of gestation (mean age 36.6 years), four women underwent a caesarean section to provide perineal protection. No complications occurred.

Only one patient had two first-trimester miscarriages, but ASCs therapy does not appear to be associated with them. According to the authors, therapy with ASCs, from the analyzed data of this small study, does not appear to affect fertility and fetal development. However, larger studies are needed to obtain more reliable information [102].

In Table 1, we summarized the literature reviewed regarding the use of MSCs for pCD [64,68,75,93,94,96,98,103,104,105,106,107,108,109,110,111].

## 5. Conclusions

There are still many unsolved questions regarding MSCs therapy for pCD. Firstly, it is necessary to evaluate the optimal dosage of MSCs treatment and, secondly, their clinical application in CD patients with active proctitis who have been excluded from many studies is still debated. Lastly, the optimal route and modality of administration should be established and standardized.

## Figures and Tables

**Table 1:** Trial summary.

Authors	Year	*n* Patients (Missing)	Cell Type	Intervention	Time-Point	Healing (%)	Follow-Up	Recurrence %
Garcia-Olmo et al. [64]	2005	9 fistulas in 4 patients with CD: -1 PF (Suprasphincteric) -3 Rectovaginal -5 Enterocutaneous	Adipose autologous	Local injection of 3 × 10^6^ stem cells	8 weeks	6/8 (75%) -1/1 -2/3 -3/4 (1 NA)	NA	NA
Wainstein et al. [111]	2008	11 fistulas in 8 patients with CD 8 PF (Trans-sphincteric) 1 PF (Inter-sphincteric) 2 Pouch-vaginal	ASCs + Platelet-rich plasma (PRP)	Local injection of 100–120 million ASCs + PRP	21–37 months	10/11 (91%) CH 1/11 (9%) PH	37 months	0%
Garcia-Olmo et al. [75]	2009	49(1) patients (of which 14 with CD) -24 fistulas of which 7 PF + CD -25 fistulas of which 7 PF + CD	Adipose autologous	Local injection of 2 × 10^6^ of Stem cells + Fibrin glue Fibrin glue alone	8 weeks	-17/24 (70.8%) of which 5/7 (71%) with CD -4/25 (16%) of which 1/7 (14%) with CD	52 weeks	(17.6%)
Ciccocioppo et al. [103,104]	2011	10 patients with CD: -9 PF (NS) -1Enterocutaneous	Bone marrow autologous	Local injection of 1.5–3 × 10^7^	52 weeks	7/10 (70%) -6/9 -1/1	52 weeks	0%
de la Portilla et al. [106]	2012	24 (8) patients with PF in CD: 17 PF (Trans-sphincteric) 5 PF (Inter-sphincteric) 1 PF (Extra-sphincteric) 1 PF (Supra-sphincteric)	Adipose allogeneic	Local injection of 2 × 10^7^ (+4 × 10^7^)	24 weeks	8/16 (50%)	24 weeks	20.0%
Cho et al. [105]	2013	9 (1) patients with CD: -3 PF -3 PF -3 PF of which: 5 (Trans-sphincteric) 4 (Supra-sphincteric) 1 (Extra-sphincteric)	Adipose autologous	Local injection of 1 × 10^7^ or 2 × 10^7^ or 4 × 10^7^	8 weeks	3/9 (33.3%) -0/3 -2/3 -1/3	8 months	0%
Lee et al. [96,108]	2013	33 (17) patients with PF -24 PF (Trans-sphincteric) -4 PF (Inter-sphincteric) -5 PF (Extrasphincteric)	Adipose autologous	Local injection 3 × 10^7^ or 6 × 10^7^	8 weeks	27/33 (81.8%)	1 year/2 years After 1 year 23/26 (88.5%) CH After 2 years 20/24 (83.3%) CH	8 weeks:11.1% 1 year: 11.5% 2 years:16.7%
Molendijk et al. [77]	2015	21 patients (with 23 PF): -5 patients with: 3 Trans-sphincteric 1 Inter-sphincteric 1 Supra-sphincteric -5 patients with: 2 Trans-sphincteric 1 Inter-sphincteric 2 Extra-sphincteric -5 patients with: 5 Trans-sphincteric -6 patients with: 5 Trans-sphincteric 1 Inter-sphincteric 1 Superficial 1 Extrasphinteric	Bone marrow alogeneic	Local injection: -1 × 10^7^ -3 × 10^7^ -9 × 10^7^ -NaCl (Placebo)	24 weeks	-4/5 (80%) -4/5 (80%) -1/5 (20%) -2/6 (33.3%)	24 weeks	Only 1 Extrasphinteric fistula in placebo group recurred 1/23 = 0.04%
Garcia-Olmo-Guadalajara [110]	2015	10 patients with PF (7 non-CD; 3 CD) Type of PF: NS	Autologous ASCs or Allogenic ASCs or SVF	Local injection of 2–3 × 10^4^ cells	8 weeks	6/10 (60%) CH 3/10 (30%) PH Of 3 patients with CD: 1 with CD: CH 1 with CD: PH 1 with CD: NO healing	1 year 60.0% CH	40.0%
Park et al. [107]	2015	6 patients with CD: -3 PF (Suprasphincteric) -3 Fistulas: 1 PF (Suprasphincteric) 1 PF (Trans-sphincteric) 1 Rectovaginal	Adipose allogeneic	Local injection of 4.33 × 10^7^ 17 × 10^7^	34 weeks	3/6 (50%) -2/3 -1/3		NA
Panes et al. [68,94]	2016	171(41) patients with PF: -88 (19) PF -83 (22) PF Type of PF: NS	Adipose allogeneic	Local injection: -12 × 10^7^ -Placebo	24 weeks	53/107(49.5%) 36/105(34.3%)	52 weeks	(25%) (44.1%)
Dietz et al. [98]	2017	12 patients with PF in CD: 8 PF (Trans-sphincteric) 3 PF (Inter-sphincteric) 1 PF (Suprasphincteric)	Adipose autologous	Local injection of 2 × 10^6^ on GoreBioA plug	26 weeks	10/12 (83.3%)		NA
Herreros et al. [93]	2019	45 patients (52 fistulas): 18 PF in CD (NS) 24 PF non-CD (NS) 7 Rectovaginal 1 Urethrorectal 1 Sacral 1 Hidradenitis suppurativa	Autologous ASCs or Allogenic ASCs or SVF	Local injection of around 48 million cells: SVF (31/52) 60% Allo-ASCs (12/52) 23% Auto-ASCs (9/52) 17%	26 weeks	49/52 (94.2%) PH–CH 24 /52 (46.2%) CH Of 18 CD fistulas: 10/18 (55.5%) CH	1 year	0%
Barnhoorn et al. [109]	2020	21(5) patients with PF: -5(1) patients -5 (1) patients -5 patients -6 (3) patients Type of PF: NS	Bone marrow alogeneic	Local injection: -1 × 10^7^ -3 × 10^7^ -9 × 10^7^ -Placebo	4 years	-3/4(75%) -4/4(100%) -1/5(20%) -0/3(0%)	4 years	Only in placebo-group all fistulas recurred after 4 year.

NA: not analyzed; CD: Crohn Disease; NS: not specified; SVF: Stromal Vascular Fraction; PH: Partial Healing; CH: Complete Healing; PF: Perianal Fistula; PRP: Platelet-rich plasma.
